# PLVAP mediates the regulation of the tumour microenvironment in early‐stage lung adenocarcinoma

**DOI:** 10.1002/ctm2.70532

**Published:** 2025-12-22

**Authors:** Linshan Xie, Mengting Sun, Yujie Zheng, Zezhong Yu, Hui Kong, Jinjie Yu, Shaohua Lu, Yong Zhang, Jie Hu, Hongyi Xin, Jian Zhou, Xiangdong Wang, Charles A. Powell, Fred R. Hirsch, Chunxue Bai, Yuanlin Song, Jun Yin, Dawei Yang

**Affiliations:** ^1^ Department of Pulmonary and Critical Care Medicine Zhongshan Hospital (Xiamen) Fudan University Xiamen China; ^2^ Department of Pulmonary and Critical Care Medicine Zhongshan Hospital Fudan University Shanghai China; ^3^ Department of Pathology Zhongshan Hospital Fudan University Shanghai China; ^4^ Department of Thoracic Surgery Zhongshan Hospital Fudan University Shanghai China; ^5^ Department of Thoracic Surgery Shanghai Geriatric Medical Center Shanghai China; ^6^ Global Institute of Future Technology Shanghai Jiao Tong University Shanghai China; ^7^ Shanghai Engineer and Technology Research Center of Internet of Things for Respiratory Medicine Shanghai China; ^8^ Shanghai Key Laboratory of Lung Inflammation and Injury Shanghai China; ^9^ Shanghai Respiratory Research Institution Shanghai China; ^10^ Shanghai Institute of Clinical Bioinformatics Shanghai China; ^11^ Shanghai Engineering Research for AI Technology for Cardiopulmonary Diseases Fudan University Shanghai Medical College Shanghai China; ^12^ Department of Pulmonary, Critical Care and Sleep Medicine Icahn School of Medicine at Mount Sinai New York New York USA; ^13^ Tisch Cancer Institute Center for Thoracic Oncology Mount Sinai Health System New York New York USA

**Keywords:** PLVAP, single‐cell RNA sequencing, tip cells, tumour endothelial cells

## Abstract

**Background:**

Early tumour vascular invasion contributes to cancer progression. Tip cells, a subset of tumour endothelial cells, significantly decline after anti‐angiogenic therapy. However, their behaviour and the roles of their signature genes during early invasion are incompletely understood.

**Methods:**

This study employed single‐cell transcriptomic analysis and 10x Genomics Visium spatial transcriptomics on fresh lung tissues from patients with pulmonary nodules and from *Kras^G12D^* (K) and *Kras^G12D^Tgfbr2^−/−^* (KT) mice. The role of plasma vesicle‐associated protein (PLVAP), a tip cell marker, was further examined using survival databases, immunofluorescence, in vitro co‐culture, cell migration, invasion assays and endothelial tube formation.

**Results:**

Tip cell proportions were elevated in early‐stage lung adenocarcinoma (LUAD) tissues and KT mice, with evidence suggesting they arise from capillaries type I. PLVAP expression was enriched in tumour endothelial cells, induced by TGFβ1, and negatively correlated with patient prognosis. Functionally, PLVAP promoted endothelial cell invasion, migration and angiogenesis, and regulated tumour cell invasiveness. Intercellular analysis revealed that some tip cells also expressed TGFβ1, which may act on adjacent tumour cells to enhance invasion during early tumour development.

**Conclusion:**

Tip cells increased during early LUAD progression and likely evolved from capillaries type I. Their marker PLVAP was associated with poor prognosis and pro‐invasive endothelial behaviour. Tumour‐secreted TGFβ1 upregulated PLVAP in endothelial cells, promoting angiogenesis and tumour invasion. Additionally, tip‐cell‐derived TGFβ1 may further stimulate tumour aggressiveness, highlighting a reciprocal interaction that contributes to early tumour progression.

**Key points:**

- Tip cells expand during early LUAD progression and likely originate from capillary type I endothelial cells.
- Tumour‐derived TGFβ1 induces PLVAP expression in endothelial cells, linking tumour signals to vascular activation.
- PLVAP enhances endothelial cell migration, invasion and angiogenic capacity.
- Endothelial PLVAP promotes tumour cell invasiveness, revealing a reciprocal endothelial‐tumour interaction that drives early tumour progression.

## Graphical abstract

This study identifies endothelial PLVAP as a prognostic marker in LUAD, linking tumour‐derived TGFβ1 to PLVAP‐driven vascular remodelling and tumour invasiveness. It reveals reciprocal TGFβ1‒PLVAP crosstalk between tip cells and tumours, proposing vascular‐targeted strategies to disrupt early metastatic niches.

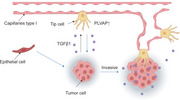

## 1 INTRODUCTION

The vascular network within tumours promotes growth by transporting oxygen and nutrients, and serve as a channel for tumour cell dissemination.^1^ One of the essential traits enabling cancer development is its capacity to induce angiogenesis. The presence of obvious signs of tumour vasculature in early pure ground‐glass pulmonary nodular lesions will significantly increase the probability of a malignant solid component in subsequent dynamic follow‐up,^2^ implying that early tumour vascular invasion is closely related to tumour progression. Endothelial cells (ECs), forming a single‐cell layer lining the interior of blood and lymphatic vessels, are key regulators of gas and metabolite exchange between the vasculature and tissues. They also contribute to the control of haemodynamics, coagulation, angiogenesis and inflammatory responses.^3^, ^4^ Tumour endothelial cells (TECs) serve as essential constituents of the tumour microenvironment (TME) that facilitate tumour growth and metastasis, independent of the cancer's organ of origin.^5^ Anti‐angiogenic therapy (AAT) is used clinically to combat a range of cancer types, however, its application has been restricted by suboptimal efficacy and the development of drug resistance. Recently, single‐cell RNA sequencing (scRNA‐seq) analyses of TECs have revealed novel mechanistic insights, highlighting promising targets for AAT.

During the process of vascular sprouting associated with tumours and other diseases, ECs can differentiate into quiescent cells, proliferating stalk cells and migrating tip cells. In a single‐cell transcriptomic analysis, Goveia et al. discovered that tip cells and stalk cells were only detected in tumour tissues. Tip cells express signature genes associated with EC migration, stromal remodelling and vascular endothelial growth factor (VEGF) signalling. Nonetheless, fewer than 10% of TECs in human lung cancer exhibit these features.^6^ As a result, scRNA‐seq data are able to outline EC heterogeneity and its translational relevance at the single‐cell level.

Mutations in the *RAS* oncogene are highly prevalent in human cancers, with *KRAS* accounting for the majority of *RAS* alterations.^7^ Overall, *KRAS* mutations are present in 35% of lung adenocarcinoma (LUAD).^8^ Conditional knock‐in mice expressing *Kras^G12D^* have previously been reported to be widely utilised to explore the pathophysiological consequences of oncogenic *RAS* expression in different tissues. *Kras^LSL‐G12D^* intranasally infused into mice with AdCre, which recognise the shear enzyme activity of specific gene sequences develops in situ spontaneous lung cancers, and has been demonstrated as a reliable experimental model for non‐invasive LUAD.^9^ Early investigations revealed that reduced expression of *Tgfbr2* in lung cancer cells promotes cellular invasiveness, indicating its potential role as a trigger in the early stages of tumour development or dissemination.^10^ *Tgfbr2* deficiency in lung epithelial cells (EPs) induces the transformation of non‐invasive *Kras* mutant tumours to an invasive form.^11^ However, different from the model of *Kras* induction in combination with *Lkb1*, *p53* and *Hif2a* knockdown, tumours in the *Kras^G12D^Tgfbr2^−/−^* (KT) model develop more slowly, similar to LUAD that manifest clinically as partially solid lung nodules.^12^

The molecular structure of plasma vesicle‐associated protein (PLVAP) consists of a short intracellular structural domain, a single transmembrane structural domain and a large extracellular structural domain.^13^ PLVAP is usually considered endothelial‐specific and plays a critical role in the formation of fenestral diaphragms and stomatal diaphragms within fenestrated and sinusoidal ECs.^14^ Involved in modulating vascular permeability under basal conditions, PLVAP also influences leukocyte transendothelial migration and angiogenic processes.^15^, ^16^ PLVAP is thought to be important in many diseases because of the ability to control the transport of soluble factors across the plasma‒interstitial barrier.^17^ *PLVAP* expression is significantly upregulated in ECs of tumour tissues from various organs, including the liver, lung, kidney, pancreas, stomach, colon, small intestine, breast, brain, ovary, uterus, prostate, skin and lymph nodes.^18^, ^19^ These observations suggest that PLVAP may be a new potential target in AAT.

However, studies on the function of PLVAP during early tumour progression are still extremely limited, especially in early‐stage LUAD which is rarely reported. The objective of the present work is to explore EC diversity during tumour progression on the basis of single‐cell transcriptomics, as well as to validate the mechanism that mediates the transformation of normal ECs into TECs by regulating *PLVAP* expression in ECs, and the related functions of the *PLVAP* gene in the TME.

## 2 METHODS

### 2.1 Data collection

We downloaded the LUAD cohort single‐cell transcriptome data HRA005794 from GSA‐Human,^20^ which contained 11 samples with CT imaging manifestations of sub‐solid pulmonary nodules (11 tumour tissues and 10 paracancerous tissues). Specifically, nine tumours harbour EGFR mutations (including six L858R and three exon 19 deletions), one tumour carries KRAS G12D mutation and one case involves RET fusion.

### 2.2 Animal models

*Kras^LSL‐G12D^* mice and *Tgfbr2^flox/flox^* mice were purchased from Jiangsu Jicui Pharmachem Biological Technology Company Limited. *Kras^LSL‐G12D^Tgfbr2^flox/flox^* mice were obtained from the identification of *Kras^LSL‐G12D^* mice and *Tgfbr2^flox/flox^* mice by crossbreeding. Mice received standard diet and water under environmental conditions of about 25°C temperature and 50% humidity. Lung cancer models were constructed by intranasal injection of 2.5 × 10^7^ pfu AdCre‐GFP into mice at 6‒8 weeks of age. After a duration of 15 weeks, mice were humanely sacrificed by spinal dislocation, and fresh lung tissues were collected for analysis.

### 2.3 Single‐cell transcriptome data analysis

The scRNA‐seq data were dimensionalised, clustered and visualised using Seurat (v 4.3.0) following standard workflows in R (v 4.3.2). Specifically, raw single‑cell data were first filtered to remove low‑quality cells (features >300 and <7000; counts <100 000; mitochondrial gene percentage <10%). Filtered counts were used directly to identify the top 2000 variable genes. Following normalisation that accounted for mitochondrial proportion, Principal component analysis (PCA) analysis was executed on variable genes, selecting the initial 18 principal components. To integrate datasets across multiple samples and correct for batch effects, we applied ‘Harmony’ function. Clustering used the Louvain algorithm (resolution = .4), and cell types were annotated by canonical marker genes.

To characterise the developmental trajectory between EC subpopulations, trajectory analysis was performed in this study by monocle2 (v 2.18.0). Seurat objects were converted to CellDataSet; genes expressed in ≥10 cells were retained, and ‘DDRTree’ was used for dimension reduction. Pseudotime ordering was performed with ‘orderCells’, and branch‑specific genes were identified via ‘BEAM’. Calculations were performed using default parameters.

Genes unique to each cluster were identified using the ‘FindAllMarkers’ function, with adjusted *p*‐values determined by the Wilcoxon rank‐sum test. The ‘FindMarkers’ function was used to identify differentially expressed genes (DEGs), applying the Wilcoxon rank‐sum test with parameters min.pct = .25 and logfc.threshold = 1.0. Genes with an adjusted *p*‐value <.05 were considered significant. Shared DEGs across multiple datasets were then designated as tip cell signature genes.

Gene Ontology (GO) enrichment analysis was conducted using the ‘clusterProfiler’ package in R, with dependencies on ‘enrichGO’ and the annotation databases ‘org.Mm.eg.db’ and ‘org.Hs.eg.db’. We used ‘CellChat’ to infer the intercellular interaction network between tip cells and EPs.

### 2.4 Spatial transcriptome analysis

To further investigate the spatial location of single‐cell clusters in the TME of early‐stage LUAD, we used the package ‘stereoscope’ to de‐convolve spatial transcriptomic data based on our scRNA‐seq data.

### 2.5 Cell culture

HUVEC, A549 and LKR cells were all cultured at 37°C in a 5% CO_2_ incubator. A549 cells were kindly provided by Prof. Xiangdong Wang's group at Zhongshan Hospital, Fudan University. HUVECs were purchased from Zhongqiao Xinzhou Biotechnology Co., Ltd. LKR cell lines were established by serial passaging of LUAD tissues isolated from Kras^G12D^ mutant mice. Using the CRISPR‐Cas9 system, we generated LKR Tgfbr2 KO cells by knocking out the Tgfbr2 gene in the parental Kras^G12D^ Tgfbr2^+/+^ LUAD cell line.

HUVECs were seeded in six‐well plates and transfected at ∼50%–70% confluence. For siRNA transfection (siRNA sequences in Table S1), 5 µL of 10 µM siRNA and 5 µL Lipofectamine RNA iMAX were each diluted in 250 µL Opti‐MEM, mixed and incubated at room temperature for 15 min. After medium replacement with 2 mL ECM, complexes were added and cells incubated at 37°C for 48 h.

HUVEC cells were seeded into six‐well plates, and when the cells were adherent to the wall and the density was around 50%, the medium of HUVEC was replaced, and 1 mL of medium was added to each well first, and shRNA transfection was performed (shRNA sequences in Table S1). HUVEC cells were transduced with shRNA lentiviral particles in endothelial cell medium (ECM) containing 2.5 µg polybrene (10 mg/mL). The volume of viral supernatant added was determined based on the viral titre and the desired multiplicity of infection. The HUVEC were placed in the incubator at 37°C for 6 h, and then 1 mL of ECM was added to each well, incubated at 37°C for 24 h, and the ECM was replaced. The transfection efficiency of the transfected cells was observed under the fluorescence microscope after 48 h for subsequent experiments.

### 2.6 Invasion, migration assay

BD Matrigel (Corning, 354234) was added to the upper chamber of transwell with 50 µL (300 µg/mL) and incubated at 37°C for 1 h. The upper chambers for migration experiments did not require the addition of BD Matrigel. The EC invasion and migration assays were performed by adding serum‐containing medium to the bottom chambers, and the upper chambers were seeded with 1 × 10^5^ of HUVEC, incubated at 37°C incubator for 24 h, fixed staining and then the chambers were put under the microscope 10× eyepiece for observation. Tumour cell invasion and migration assays were performed by seeding the bottom chamber with 6 × 10^4^ HUVEC 24 h before the experiment and incubating it in the incubator at 37°C. After Matrigel coagulation, the upper chamber was seeded with 5 × 10^4^ A549 cells, and incubated in the incubator at 37°C for 24 h, and the subsequent staining was the same as that of the EC invasion and migration.

### 2.7 Endothelial cell tube‐forming assay

Coat 96‐well plates with 50 µL (300 µg/mL) of BD Matrigel and incubate for 45 min at 37°C.

Add 100 µL of ECM containing 1 × 10^4^ HUVEC to each well and incubate in the incubator at 37°C for 6 h. Place the 96‐well plates under a microscope 40× eyepiece for observation.

### 2.8 TGFβ1 stimulation of endothelial cells assay

HUVEC cells were seeded into six‐well plates, and when the cells were adherent to the wall and the density was around 50%‒60%, TGFβ1 (Sino Biological, 10804‐HNAC) was added for stimulation. According to the TGFβ1 final concentration of 5, 10, 20 and 40 ng/mL, a certain amount of TGFβ1 solution was added to each six‐well plate, and incubated in a 37°C incubator for 12‒48 h.

### 2.9 Fluorescence quantitative PCR

After RNA extraction was completed, cDNA was produced via reverse transcription with PrimeScript RT Master Mix (Takara, RR036A). After gentle mixing, reverse transcription was performed under the following conditions: 37°C for 15 min, followed by 85°C for 5 s, then stored at 4°C. The qPCR primers (Table S2) were designed according to the experimental requirements, PCR reaction solution was assembled according to TB Green Premix Ex Taq II (Tli RNaseH Plus) guidelines, with fluorescence qPCR conducted post‐mixing.

### 2.10 ELISA

Take out the mouse TGFβ1 ELISA Kit (Xinbosheng, EMC107b) from the refrigerator 20 min in advance to equilibrate to room temperature. Perform the ELISA procedure according to the steps in the instruction manual. Finally, measure the OD_450_ value (within 3 min) in an instant assay in an enzyme marker.

### 2.11 Immunofluorescence staining

To complete the immunofluorescence staining, primary antibodies of PLVAP (abcam, ab27853) and CD31 (GeneTex, GTX34489) were sequentially used, followed by secondary antibody incubation. Finally, the nuclei were stained (Servicebio, GDP1025). The slides were stained by scanning to generate multispectral images.

### 2.12 Statistical analyses

All statistical analyses were carried out using GraphPad Prism software (version 9.0). Quantitative data were expressed as the mean ± standard error of the mean. To assess differences between two independent groups, an unpaired two‐tailed Student's *t*‐test was applied. For comparisons involving more than two groups, one‐way analysis of variance was performed. Kaplan–Meier survival curves were generated, and statistical differences were evaluated using the log‐rank test. *p*‐Value <.05 was considered statistically significant. Each experiment was independently repeated at least three times to ensure reproducibility.

## 3 RESULTS

### 3.1 Single‐cell transcriptomics reveals altered endothelial cell subpopulations in early‐stage lung adenocarcinoma

**FIGURE 1:**
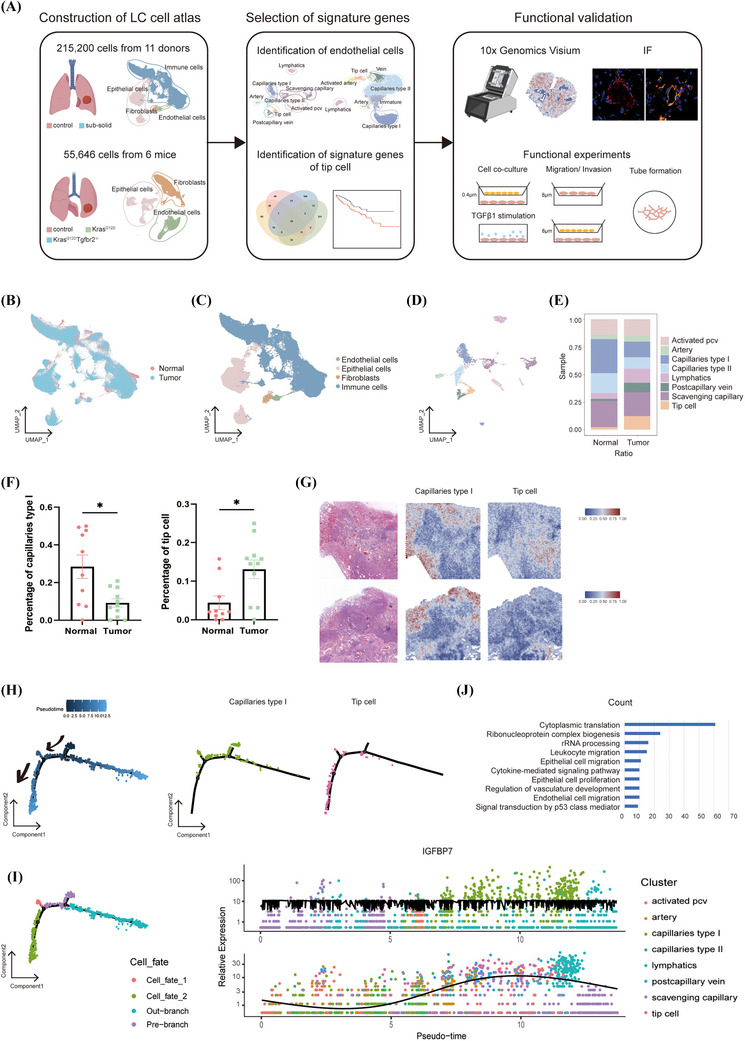
Single‐cell transcriptomics reveals altered endothelial cell subpopulations in early‐stage lung adenocarcinoma. (A) Schematic of study workflows. (B) Uniform Manifold Approximation and Projection (UMAP) plot of 215 200 single cells coloured according to sample source. (C) UMAP plot of cells coloured according to cell type. (D) UMAP plot of 2020 endothelial cells coloured according to cell subpopulation. (E) Percentage of each endothelial cell subpopulation in each sample histogram. (F) Percentage of capillaries type I and tip cells in each group. (G) Spatial visualisation of capillaries type I and tip cells in lung adenocarcinoma analysed by 10x Genomics Visium. (H) Left: cells were ordered along the pseudotime trajectory. Darker colours indicated earlier pseudotime stages, and lighter colours represented later stages. Arrows denoted the potential differentiation direction from capillary type I cells towards TIP cells. Right: pseudotime trajectories of capillary type I and TIP cell clusters were shown by cell type. (I) Left: distribution of cell fate along the pseudotime trajectory. ‘Pre‐branch’ represented the pseudotime starting point, while ‘Cell fate 1’ and ‘Cell fate 2’ denoted two distinct differentiation outcomes, and ‘Out‐branch’ referred to cells outside this trajectory. The results suggested that during endothelial differentiation, part of the capillary type I population maintained its phenotype, while another subpopulation differentiated towards TIP cells. Right: expression analysis further showed that IGFBP7 was markedly upregulated in tip cells. (J) Gene Ontology (GO) pathway enrichment analysis of tip cell populations.

To profile the TME of early‐stage LUAD, we compiled a single‐cell atlas using 215 200 cells from 11 patients with sub‐solid pulmonary nodules on clinical CT imaging (Figure 1A,B). The cells were classified into four types based on tissue type‐specific markers: ECs, EPs, fibroblasts and immune cells (Figure 1C). To further explore the heterogeneity of TECs, we again performed unsupervised cluster analysis on 2020 EC populations. Based on the top 50 marker genes of various endothelial subsets reported by Goveia et al.,^6^ we applied the AddModuleScore function to score each cell type. Eight EC subpopulations were identified as activated pcv, artery, capillaries type I, capillaries type II, lymphatics, postcapillary vein, scavenging capillary and tip cell (Figure 1D). We compared the proportions of tip cell and capillaries type I in tumour and paracancerous samples, in which tip cell increased in tumour tissues and capillaries type I decreased in tumour tissues (Figures 1E,F and S1). Meanwhile, we performed spatial transcriptomics analysis of Formalin‐fixed, paraffin‐embedded (FFPE) samples from two of these patients, using the signature genes of tip cell and capillaries type I as references to infer the distribution locations of tip cell and capillaries type I in early‐stage LUAD tissues. The results showed that capillaries type I was mainly distributed with paracancerous tissues. In contrast, tip cells were more enriched in tumours (Figure 1G). Pseudotime analysis of ECs indicated that tip cells might have originated from capillary type I cells. Cells were ordered along the trajectory, with darker colours representing earlier pseudotime stages and lighter colours representing later stages. The trajectory suggested a differentiation direction from capillary type I cells towards tip cells (Figure 1H). Cell fate mapping showed that part of the capillary type I population maintained its phenotype, whereas another subpopulation differentiated towards tip cells. Expression analysis further revealed that IGFBP7, a gene promoting endothelial adhesion, tube formation, vascular stabilisation and maturation,^21^ was significantly upregulated in tip cells, supporting their activated, angiogenic state (Figure 1I). Then, we performed GO pathway enrichment analysis on the increased number of tip cells during tumour progression, and found that this subpopulation was significantly upregulated in signalling pathways such as cytoplasmic translation, EP migration, cytokine‐mediated signalling pathway and EC migration (Figure 1J). This may indicate the role of capillaries type I and tip cells in tumour progression.

### 3.2 Lung adenocarcinoma mouse models to further validate the alteration of endothelial cell subpopulations during tumour progression

**FIGURE 2:**
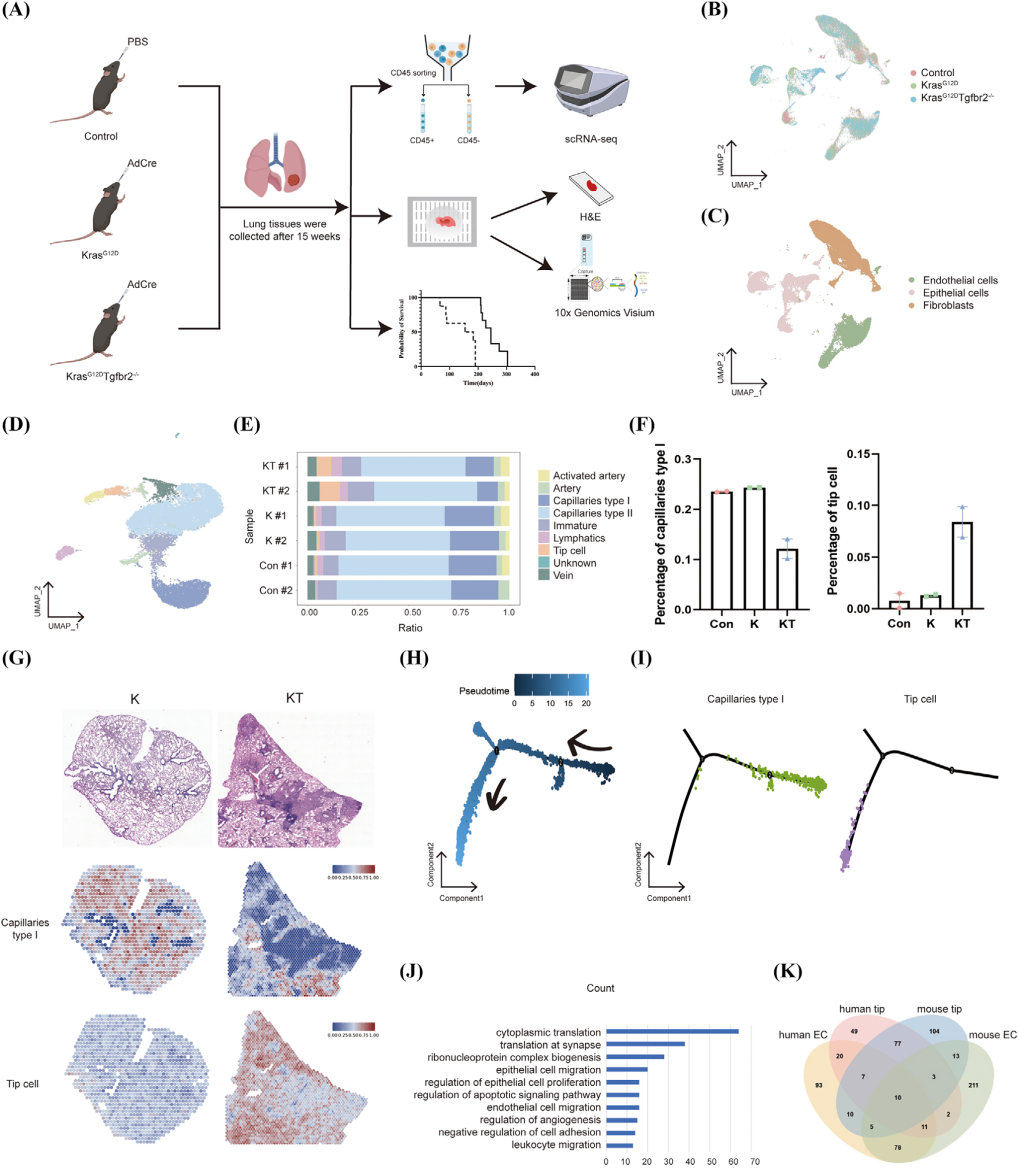
Heterogeneity of endothelial cells in mice models of lung cancer. (A) Schematic of study workflows. (B) UMAP plots of 55 646 single cells coloured according to sample source. (C) UMAP plots of cells coloured according to cell type. (D) UMAP plots of 19 048 endothelial cells coloured according to cell subpopulation. (E) Each endothelial cell subpopulation in each sample percentage histogram. (F) Percentage of capillaries type I and tip cells in each group. (G) Spatial visualisation of capillaries type I and tip cell in K and KT analysed by 10x Genomics Visium. (H) Pseudotime ordering of endothelial cells from mouse lung samples showed the sequential distribution of different subtypes. (I) Pseudotime trajectories of capillary type I and tip cell clusters in mouse samples. Arrows indicated a potential differentiation direction from capillary type I cells towards tip cells. (J) Gene Ontology (GO) pathway enrichment analysis of tip cell populations. (K) Selection of tip cell signature genes (adjusted *p* < .05 and |log_2_FC| > 1.0).

Considering the heterogeneity of clinical samples, we used *Kras^LSL‐G12D^* and *Kras^LSL‐G12D^Tgfbr2^flox/flox^* mouse models to simulate the process of tumour invasion through in situ gene‐induced mutations.^9^ Fresh lung tissues from mice were harvested 15 weeks after modelling, and in order to enrich for more ECs, we conducted CD45 flow sorting of single‐cell suspensions, and the obtained CD45+ and CD45‒ single‐cell suspensions were subjected to subsequent single‐cell sequencing, respectively (Figure 2A). First, we analysed 55 646 CD45‒ cells derived from control, *Kras^G12D^* (K) and *Kras^G12D^Tgfbr2^−/−^* (KT) and identified three cell types, namely, ECs, EPs and fibroblasts (Figure 2B,C).

To further explore the heterogeneity of individual subpopulations in ECs, we again ran unsupervised cluster analysis on 19 048 EC populations. Nine EC subpopulations were identified as activated artery, artery, capillaries type I, capillaries type II, immature, lymphatics, tip cell, vein and unknown (Figure 2D). Similarly, we compared the proportions of tip cell and capillaries type I in the three groups. It was found that the proportion of tip cell increased in the KT group and the proportion of capillaries type I decreased in the KT group (Figure 2E,F). We performed spatial transcriptomics analysis of K and KT mice and used the signature genes of tip cell and capillaries type I as references to infer the distribution locations of tip cell and capillaries type I in K and KT mice. A notably higher density of capillaries type I was observed in the K group compared to the KT group. Tip cells were significantly more abundant in the KT group and more enriched around the tumours (Figure 2G). Consistent with the human data, a similar pseudotime trajectory was observed in mouse lung samples (Figure 2H,I), further supporting the potential transition from capillary type I cells to tip cells. These findings collectively strengthen the evidence for a lineage relationship between capillary type I and tip cells in the endothelium. Then, we performed GO pathway enrichment analysis on the increased tip cells during tumour progression and found that this subpopulation was significantly upregulated in signalling pathways such as cytoplasmic translation, EP migration, regulation of apoptotic signalling pathway, EC migration and regulation of angiogenesis (Figure 2J). This may indicate a role for capillaries type I and tip cells in tumour progression.

### 3.3 Selection of signature genes of tip cell associated with tumour progression

**FIGURE 3:**
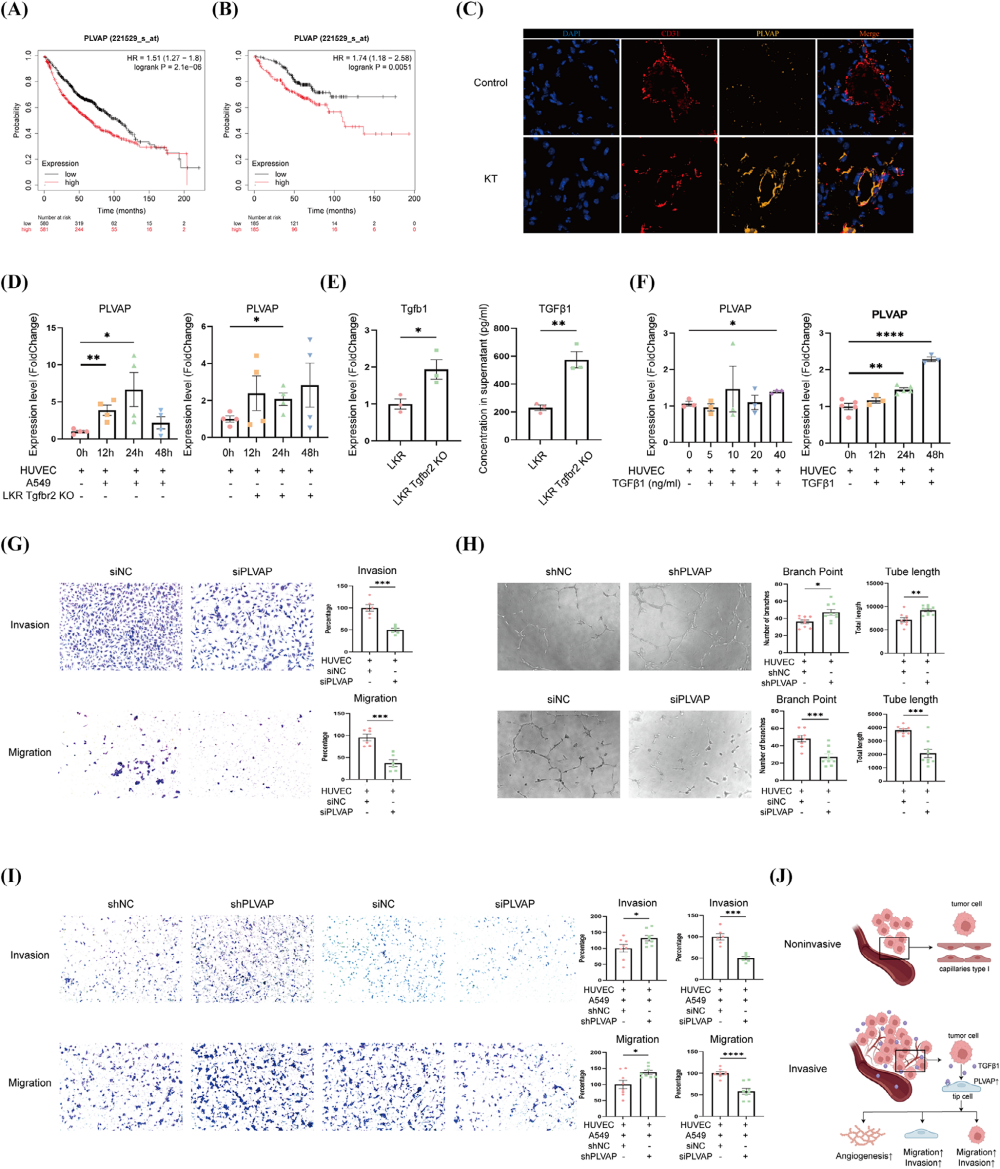
Tumour cells promote plasma vesicle‐associated protein (PLVAP) expression in endothelial cells by secreting TGFβ1. (A) Survival prognosis of all stages of lung adenocarcinoma. (B) Survival prognosis of stage I lung adenocarcinoma. (C) Representative immunofluorescence staining images of control mice and KT mice. (D) PLVAP expression levels on HUVEC after co‐culture with tumour cells. (E) The expression level of TGFβ1 in tumour cells and supernatants. (F) PLVAP expression levels on HUVEC after TGFβ1 stimulation at different concentrations and different times. (G) Invasion and migration assays on HUVEC after siRNA knockdown of PLVAP on HUVEC. (H) siRNA knockdown of PLVAP on HUVEC and shRNA elevation of PLVAP on HUVEC, the tube‐forming ability of HUVEC. (I) Invasion and migration assays of A549 in HUVEC co‐cultured with A549 with elevated or knocked down PLVAP. (J) Diagram of PLVAP‐mediated regulation of the tumour microenvironment in lung adenocarcinoma. ^*^ *p* < .05; ^**^ *p* < .01; ^****^ *p* < .0001.

According to the results of the human and mouse models, we expected to identify the potential target genes of tip cells that play important roles in tumour progression. We took the intersection of human tip cell over capillaries type I differential genes, human EC signature genes, mouse tip cell over capillaries type I differential genes, and mouse EC signature genes, and obtained a total of 10 signature genes (Figure 2K). Next, we further analysed these 10 tip cell signature genes. As tip cells are mainly enriched in tumour tissues, and the expression of tip cell signature genes correlates with poorer tumour prognosis.^6^ Based on multiple reports that the tip EC phenotype—characterised by enhanced angiogenic sprouting and immunosuppressive activity—generally linked to poorer prognosis in Non‐small cell lung cancer (NSCLC) and other solid tumours,^22^ we hypothesised that within our tip cell signature there exists a subset of genes whose high expression directly portends poor prognosis. To identify prognostic tip‐cell‐associated genes, we performed survival analysis using the Kaplan–Meier Plotter database based on LUAD datasets (https://kmplot.com/analysis/). The expression cutoff was set to the median, and overall survival was analysed across different disease stages. We found that high expression of the PLVAP gene was associated with a poorer overall survival rate, while all other characteristic genes were positively correlated with survival rate. Given this unique adverse prognostic association, we focused our functional studies on PLVAP (Figures 3A,B and S2). Although bulk RNA‐seq datasets mainly reflect whole tumour tissues, PLVAP is specifically expressed in TECs and not in EPs. Consistently, LUAD scRNA‐seq datasets confirmed PLVAP restriction to endothelial subsets.^23^, ^24^ Therefore, the prognostic associations identified in bulk datasets reliably reflect endothelial PLVAP expression. Nevertheless, we acknowledged that when assessing complex stromal or immune cell populations, gene set‐based approaches would have been more appropriate, which represented a limitation of this study.

### 3.4 Tumour cells stimulate PLVAP expression in endothelial cells

We first demonstrated by immunofluorescence staining that PLVAP co‐localised with CD31, a marker gene for ECs, and that PLVAP expression was elevated in the vasculature of KT mice (Figure 3C). There is still limited research on what factors stimulate increased PLVAP expression on ECs. We found significantly enhanced PLVAP expression on ECs using human (A549) or mouse LUAD tumour cell lines (LKR Tgfbr2 KO) co‐cultured with HUVEC (Figure 3D).

Based on the previous results the proportion of tip cells was found to be significantly increased after *Tgfbr2* deletion (KT mice). We knocked down *Tgfbr2* on *Kras* mutant LUAD cell line (LKR) by in vitro and found high expression of TGFβ1 in tumour cells and supernatant (Figure 3E). Next, stimulation of ECs using TGFβ1 revealed that PLVAP on ECs increased with stimulation time and concentration gradient (Figure 3F). Thus, it is suggested that the regulation of PLVAP expression on ECs may be related to the secretion of TGFβ1 by tumour cells.

### 3.5 Function of PLVAP on endothelial cells

The exploration of the effects of elevated PLVAP on ECs in the TME is still very limited. We knocked down PLVAP on HUVEC using siRNA and found that both invasion and migration of ECs were significantly attenuated (Figure 3G). Subsequently, we used shRNA to elevate PLVAP on HUVEC to simulate the elevated expression of PLVAP in ECs stimulated by tumour cells, and found that the tube‐forming ability of HUVEC was significantly enhanced after elevation of PLVAP. After knocking down PLVAP with siRNA, the tube‐forming ability of HUVEC was significantly attenuated. The outcomes suggested that PLVAP in ECs was involved in the regulation of angiogenesis (Figure 3H). Then, we co‐cultured HUVEC with A549 and found that HUVEC‐expressed PLVAP was able to regulate tumour cell invasion and migration (Figure 3I). These observations pointed to the conclusion that PLVAP on ECs exerted a significant influence on the LUAD microenvironment (Figure 3J).

### 3.6 Tip‐cell‐mediated tumour progression

**FIGURE 4:**
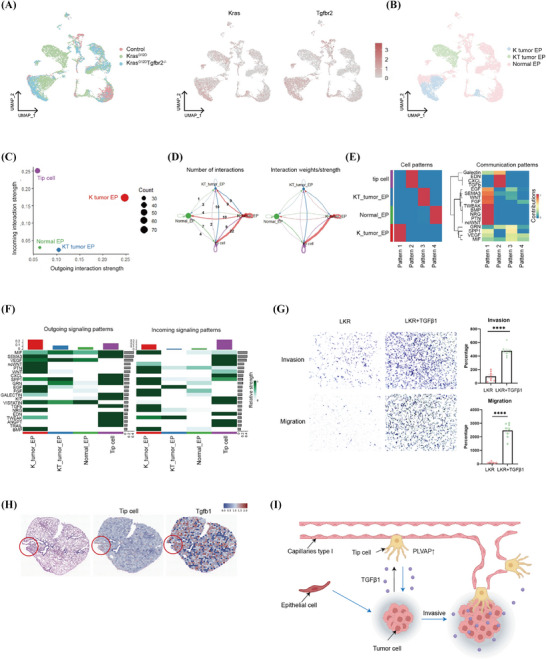
Tip cells expressing plasma vesicle‐associated protein (PLVAP) enhance tumour cell invasiveness. (A) UMAP plots of 14 226 epithelial cells coloured according to sample source, *Kras* gene and *Tgfbr2* gene expression. (B) UMAP plots coloured according to cell type. (C) The potential intercellular communication strength of each cluster, in both directions, was analysed via CellChat. (D) The circle diagram displays the variation in both the quantity and intensity of interactions between tip and epithelial cells. (E) Non‐negative matrix factorisation (NMF) recognises cellular communication patterns. (F) Summary of outgoing and incoming signalling patterns across control, K and KT samples. (G) Invasion and migration assays of LKR cells after TGFβ1 stimulation. (H) Spatial visualisation of tip cell and Tgfb1 in *Kras* mouse model analysed by 10x Genomics Visium. (I) Diagram of tip‐cell‐mediated tumour progression.

Furthermore, we expected to explore the interaction of PLVAP‐expressing tip cell with tumour cells during tumour progression. First, we analysed 14 226 EPs, which were classified into normal EP, K tumour EP and KT tumour EP according to their origin and the level of expressing *Kras* and *Tgfbr2* (Figure 4A,B). Then, we found that K tumour EP was the center of interaction by CellChat analysis, and there were more interactions with tip cells (Figure 4C,D). Meanwhile, CellChat results inferred that tip cell expressing Tgfb acted on K tumour EP (Figure 4E,F), and we also found in in vitro experiments that stimulating LKR cells using TGFβ1 could significantly enhance tumour cell invasion and migration (Figure 4G). In our 10x Genomics Visium spatial transcriptomic analysis of Kras mouse models, we demonstrated that tip cells express Tgfb1 (Figure 4H). Therefore, we deduced that a small number of tip cells would express TGFβ1 acting on tumour cells at the early stage of tumour to enhance their invasiveness (Figure 4I).

## 4 DISCUSSION

Overall, we demonstrated that tip cells, a subpopulation of TECs, are significantly elevated in tumour tissues by single‐cell transcriptomics and spatial transcriptomics. Specifically, we simulated the early tumour infiltration process using K and KT mouse models as a way to explore the heterogeneity of ECs during tumour invasion. Our results showed a notable elevation in the fraction of tip cells and enriched in tumour tissue during tumour infiltration from the inert stage to malignancy. This subpopulation was associated with activation of gene pathways related to migration of epithelial and ECs, along with modulation of angiogenic processes, suggesting that tip cells may be involved in tumour progression. Previous studies have found that tip cells are distributed at the anterior tip of vascular branches in vascular sprouting and are regulated by VEGF, NOTCH signalling and the glycolysis regulator PFKFB3,^3^, ^25^ which is tightly linked to the process of neovascularisation. The proportion of tip cells in tumours is significantly downregulated after treatment with anti‐angiogenic drugs.^26^

Our pseudotime analysis of ECs in clinical pulmonary nodules as well as early‐stage LUAD in mice revealed that tip cells may evolve from capillaries type I, which express genes associated with immunoregulation.^6^ We explored the factors that promote the transformation from capillaries type I to tip cell during tumour progression by differential gene analysis, and found that PLVAP was upregulated in tip cells in both human and mice, and negatively linked to survival rates among patients diagnosed with LUAD. PLVAP is considered as a marker for TECs in most tumours.^24^ Beyond LUAD, PLVAP has been implicated in multiple tumour types via distinct upstream regulators and downstream pathways. In glioblastoma, single‐cell analyses reveal PLVAP enrichment in contrast‐enhancing tumour capillaries, where it mediates blood–brain barrier disruption and vascular leakage.^27^, ^28^, ^29^ In cholangiocarcinoma, PLVAP is upregulated through a DKK1/CKAP4/PI3K‑Akt axis, correlates with micro‐vessel density and poor prognosis.^30^ Moreover, pancreatic EC atlases identify PLVAP as a key permeability gene downstream of NKX2‑3, contributing to islet capillary specialisation and barrier function.^31^ Meanwhile, targeting PLVAP can effectively inhibit tumour growth in cholangiocarcinoma, pancreatic cancer and hepatocellular carcinoma.^30^, ^32^, ^33^ PLVAP may also participate in tumour immune regulation by modulating vascular permeability and endothelial barrier function.^17^ In tumours, high PLVAP levels are linked to abnormal vasculature that restricts effector immune cell infiltration and supports immune evasion.^34^ In hepatocellular carcinoma and glioblastoma, PLVAP⁺ vessels correlate with reduced cytotoxic T‐cell presence.^33^, ^35^ However, studies on the function of PLVAP in early tumour progression are still very limited, especially in human early‐stage LUAD, which is rarely reported. In contrast, our data in LUAD uncover a TGFβ1‐centric mechanism whereby Tgfbr2‐deficient tumour cells secrete TGFβ1 to induce PLVAP in tip‐like ECs, driving tumour invasiveness. We aimed to uncover the pro‐invasive role of PLVAP⁺ tip‐like ECs in the early progression of LUAD and to propose the TGFβ1‒PLVAP axis as a critical mechanism underlying early tumour–endothelium crosstalk. We found that PLVAP expression on ECs was significantly increased under the stimulation of LUAD cells. TGFβ1 was also found to be important in upregulating PLVAP in ECs. Tumour cells simulating the *Kras* mutation *Tgfbr2* deletion in vitro were able to highly express and hyper‐secrete TGFβ1. Previous studies have shown that TGFβ promotes ECs proliferation, migration,^36^ angiogenesis and angiogenic factors expression such as VEGFA.^37^ Moreover, recent single‐cell analyses in ovarian and lung cancers demonstrate that tip‐like ECs exhibit induced TGFβ1 expression in response to tumour‐derived cues, rather than spontaneous production.^38^ This supports a reinforcing feedback mechanism was involved PLVAP and TGFβ1, as well as functional divergence between tumour‐ and tip‐cell‐derived TGFβ1. This may explain that during tumour progression tumour cells promote tumour growth and metastasis by secreting TGFβ1 and activating neighboring ECs to express PLVAP. Further, we found that the upregulated PLVAP gene in TECs could regulate the invasion and migration of ECs, enhance angiogenesis, which in turn supports tumour cell invasiveness and movement, thus promoting tumour progression. Meanwhile, our findings indicate that during the initial phase of tumour infiltration, a subset of tip cells expressed TGFβ1, which promoted tumour cell invasiveness. This provides new evidence to support the therapeutic target of PLVAP that targets TECs. Therapeutically, PLVAP inhibition using monoclonal antibodies, antibody‒drug conjugates, or peptide/nanobody blockers offers the potential to selectively disrupt pathological angiogenesis while preserving normal vasculature. Preclinical studies demonstrate efficacy in vascular normalisation and enhanced chemotherapeutic delivery.^22^, ^33^, ^39^, ^40^ Clinically, patient stratification by PLVAP expression and integration with anti‐VEGF or immune checkpoint therapies could increase efficacy.^41^, ^42^ In future trials, patients with high tip cell signatures may benefit most from PLVAP blockade, and imaging biomarkers such as dynamic contrast‐enhanced magnetic resonance imaging may help assess vascular responses and stratify patients according to treatment benefit.^43^

This research, while supported by comprehensive in vivo and in vitro data, is subject to several limitations. The small sample size of LUAD cases might not fully represent the entire heterogeneity of patient tumours, and the *Kras^G12D^Tgfbr2^2^/^2^* mouse model, while useful for dissecting specific genetic effects, cannot fully replicate the complexity of human TMEs and immune responses. Moreover, this study is the focus on TGFβ1 without comprehensive analysis of other cytokines that may be upregulated after *Tgfbr2* loss, such as IL‐6, CXCL1 and CCL5, which are also involved in immune suppression, angiogenesis and stromal remodelling.^44^, ^45^, ^46^ Although our co‑culture assays and analyses of public human scRNA‑seq datasets reinforce the key findings, validation in prospective clinical cohorts will be essential to determine PLVAP's true value as a diagnostic marker and therapeutic target.

## AUTHOR CONTRIBUTIONS

Linshan Xie and Mengting Sun contributed equally to this work. Yujie Zheng, Zezhong Yu, Hui Kong, Jinjie Yu, Shaohua Lu, Yong Zhang, Jie Hu, Hongyi Xin, Jian Zhou, Xiangdong Wang, Charles A. Powell and Fred R. Hirsch participated in the design, data collection and analysis. Chunxue Bai, Yuanlin Song and Jun Yin provided clinical expertise and critical revision of the manuscript. Dawei Yang conceived and supervised the study. All authors reviewed and approved the final manuscript.

## CONFLICT OF INTEREST STATEMENT

The authors declare they have no conflicts of interest.

## ETHICS STATEMENT

This study was approved by the Ethics Committee of Zhongshan Hospital, Fudan University (EC, Zhongshan Hospital) and gave its approval to the project (ethics approval no. B2022‐402R).

## Supporting information



Supporting Information

Supporting Information

Supporting Information

## Data Availability

The data that support the findings of this study are openly available in the China National Center for Bioinformation/Beijing Institute of Genomics, Chinese Academy of Sciences (GSA‐Human: HRA005794) at https://ngdc.cncb.ac.cn/gsa-human.

## References

[1] Strilic B , Yang L , Albarrán‐Juárez J , et al. Tumour‐cell‐induced endothelial cell necroptosis via death receptor 6 promotes metastasis. Nature. 2016;536(7615):215‐218. doi:10.1038/nature19076 27487218 10.1038/nature19076

[2] Le V , Yang D , Zhu Y , et al. Quantitative CT analysis of pulmonary nodules for lung adenocarcinoma risk classification based on an exponential weighted grey scale angular density distribution feature. Comput Methods Programs Biomed. 2018;160:141‐151. doi:10.1016/j.cmpb.2018.04.001 29728241 10.1016/j.cmpb.2018.04.001

[3] Potente M , Gerhardt H , Carmeliet P . Basic and therapeutic aspects of angiogenesis. Cell. 2011;146(6):873‐887. doi:10.1016/j.cell.2011.08.039 21925313 10.1016/j.cell.2011.08.039

[4] Pober JS , Sessa WC . Evolving functions of endothelial cells in inflammation. Nat Rev Immunol. 2007;7(10):803‐815. doi:10.1038/nri2171 17893694 10.1038/nri2171

[5] Hanahan D . Hallmarks of cancer: new dimensions. Cancer Discov. 2022;12(1):31‐46. doi:10.1158/2159‐8290.CD‐21‐1059 35022204 10.1158/2159-8290.CD-21-1059

[6] Goveia J , Rohlenova K , Taverna F , et al. An integrated gene expression landscape profiling approach to identify lung tumor endothelial cell heterogeneity and angiogenic candidates. Cancer Cell. 2020;37(1):21‐36. doi:10.1016/j.ccell.2019.12.001 31935371 10.1016/j.ccell.2019.12.001

[7] Prior IA , Lewis PD , Mattos C . A comprehensive survey of Ras mutations in cancer. Cancer Res. 2012;72(10):2457‐2467. doi:10.1158/0008‐5472.CAN‐11‐2612 22589270 10.1158/0008-5472.CAN-11-2612 PMC3354961

[8] Prior IA , Hood FE , hartley JL . The frequency of ras mutations in cancer. Cancer Res. 2020;80(14):2969‐2974. doi:10.1158/0008‐5472.CAN‐19‐3682 32209560 10.1158/0008-5472.CAN-19-3682 PMC7367715

[9] Borczuk AC , Sole M , Lu P , et al. Progression of human bronchioloalveolar carcinoma to invasive adenocarcinoma is modeled in a transgenic mouse model of K‐ras‐induced lung cancer by loss of the TGF‐β type II receptor. Cancer Res. 2011;71(21):6665‐6675. doi:10.1158/0008‐5472.CAN‐11‐1590 21911454 10.1158/0008-5472.CAN-11-1590 PMC5977996

[10] Borczuk AC , Kim HK , Yegen HA , et al. Lung adenocarcinoma global profiling identifies type II transforming growth factor‐beta receptor as a repressor of invasiveness. Am J Respir Crit Care Med. 2005;172(6):729‐737. doi:10.1164/rccm.200504‐615OC 15976377 10.1164/rccm.200504-615OC PMC2718552

[11] Yoo S , Sinha A , Yang D , et al. Integrative network analysis of early‐stage lung adenocarcinoma identifies aurora kinase inhibition as interceptor of invasion and progression. Nat Commun. 2022;13(1):1592. doi:10.1038/s41467‐022‐29230‐7 35332150 10.1038/s41467-022-29230-7 PMC8948234

[12] Powell CA . Case of invasive nodule, break ground glass. Am J Respir Crit Care Med. 2021;204(10):1124‐1126. doi:10.1164/rccm.202108‐1985ED 34582324 10.1164/rccm.202108-1985ED PMC8759304

[13] Stan RV , Arden KC , Palade GE . cDNA and protein sequence, genomic organization, and analysis of cis regulatory elements of mouse and human PLVAP genes. Genomics. 2001;72(3):304‐313. doi:10.1006/geno.2000.6489 11401446 10.1006/geno.2000.6489

[14] Ioannidou S , Deinhardt K , Miotla J , et al. An in vitro assay reveals a role for the diaphragm protein PV‐1 in endothelial fenestra morphogenesis. Proc Natl Acad Sci U S A. 2006;103(45):16770‐16775. doi:10.1073/pnas.0603501103 17075074 10.1073/pnas.0603501103 PMC1636530

[15] Carson‐Walter EB , Hampton J , Shue E , et al. Plasmalemmal vesicle associated protein‐1 is a novel marker implicated in brain tumor angiogenesis. Clin Cancer Res. 2005;11(21):7643‐7650. doi:10.1158/1078‐0432.CCR‐05‐1099 16278383 10.1158/1078-0432.CCR-05-1099

[16] Keuschnigg J , Henttinen T , Auvinen K , et al. The prototype endothelial marker PAL‐E is a leukocyte trafficking molecule. Blood. 2009;114(2):478‐484. doi:10.1182/blood‐2008‐11‐188763 19420356 10.1182/blood-2008-11-188763

[17] Rantakari P , Auvinen K , JÄPPINEN N , et al. The endothelial protein PLVAP in lymphatics controls the entry of lymphocytes and antigens into lymph nodes. Nat Immunol. 2015;16(4):386‐396. doi:10.1038/ni.3101 25665101 10.1038/ni.3101

[18] Madden SL , Cook BP , Nacht M , et al. Vascular gene expression in nonneoplastic and malignant brain. Am J Pathol. 2004;165(2):601‐608. doi:10.1016/S0002‐9440(10)63324‐X 15277233 10.1016/s0002-9440(10)63324-x PMC1618572

[19] Strickland LA , Jubb AM , Hongo JA , et al. Plasmalemmal vesicle‐associated protein (PLVAP) is expressed by tumour endothelium and is upregulated by vascular endothelial growth factor‐A (VEGF). J Pathol. 2005;206(4):466‐475. doi:10.1002/path.1805 15971170 10.1002/path.1805

[20] Xie L , Kong H , Yu J , et al. Spatial transcriptomics reveals heterogeneity of histological subtypes between lepidic and acinar lung adenocarcinoma. Clin Transl Med. 2024;14(2):e1573. doi:10.1002/ctm2.1573 38318637 10.1002/ctm2.1573 PMC10844893

[21] Lit KK , Zhirenova Z , Blocki A . Insulin‐like growth factor‐binding protein 7 (IGFBP7): a microenvironment‐dependent regulator of angiogenesis and vascular remodeling. Front Cell Dev Biol. 2024;12:1421438. doi:10.3389/fcell.2024.1421438 39045455 10.3389/fcell.2024.1421438 PMC11263173

[22] Kane K , Edwards D , Chen J . The influence of endothelial metabolic reprogramming on the tumor microenvironment. Oncogene. 2025;44(2):51‐63. doi:10.1038/s41388‐024‐03228‐5 39567756 10.1038/s41388-024-03228-5 PMC11706781

[23] Qian J , Olbrecht S , Boeckx B , et al. A pan‐cancer blueprint of the heterogeneous tumor microenvironment revealed by single‐cell profiling. Cell Res. 2020;30(9):745‐762. doi:10.1038/s41422‐020‐0355‐0 32561858 10.1038/s41422-020-0355-0 PMC7608385

[24] Zeng Q , Mousa M , Nadukkandy AS , et al. Understanding tumour endothelial cell heterogeneity and function from single‐cell omics. Nat Rev Cancer. 2023;23(8):544‐564. doi:10.1038/s41568‐023‐00591‐5 37349410 10.1038/s41568-023-00591-5

[25] De Bock K , Georgiadou M , Schoors S , et al. Role of PFKFB3‐driven glycolysis in vessel sprouting. Cell. 2013;154(3):651‐663. doi:10.1016/j.cell.2013.06.037 23911327 10.1016/j.cell.2013.06.037

[26] Zhao Q , Eichten A , Parveen A , et al. Single‐cell transcriptome analyses reveal endothelial cell heterogeneity in tumors and changes following antiangiogenic treatment. Cancer Res. 2018;78(9):2370‐2382. doi:10.1158/0008‐5472.CAN‐17‐2728 29449267 10.1158/0008-5472.CAN-17-2728

[27] Sharma A , Seow JJW , Dutertre CA , et al. Onco‐fetal reprogramming of endothelial cells drives immunosuppressive macrophages in hepatocellular carcinoma. Cell. 2020;183(2):377‐394.e321. doi:10.1016/j.cell.2020.08.040 32976798 10.1016/j.cell.2020.08.040

[28] Li Z , Pai R , Gupta S , et al. Presence of onco‐fetal neighborhoods in hepatocellular carcinoma is associated with relapse and response to immunotherapy. Nat Cancer. 2024;5(1):167‐186. doi:10.1038/s43018‐023‐00672‐2 38168935 10.1038/s43018-023-00672-2

[29] Ma K , Chen X , Zhao X , et al. PLVAP is associated with glioma‐associated malignant processes and immunosuppressive cell infiltration as a promising marker for prognosis. Heliyon. 2022;8(8):e10298. doi:10.1016/j.heliyon.2022.e10298 36033326 10.1016/j.heliyon.2022.e10298 PMC9404362

[30] Wang Y , Yu H , Xie X , et al. Plasmalemma vesicle‐associated protein promotes angiogenesis in cholangiocarcinoma via the DKK1/CKAP4/PI3K signaling pathway. Oncogene. 2021;40(25):4324‐4337. doi:10.1038/s41388‐021‐01844‐z 34079085 10.1038/s41388-021-01844-z

[31] Khan ST , Ahuja N , Taïb S , et al. Single‐cell meta‐analysis uncovers the pancreatic endothelial cell transcriptomic signature and reveals a key role for NKX2‐3 in PLVAP expression. Arterioscler Thromb Vasc Biol. 2024;44(12):2596‐2615. doi:10.1161/ATVBAHA.124.321781 39445426 10.1161/ATVBAHA.124.321781 PMC11594071

[32] Deharvengt S J , Tse D , Sideleva O , et al. PV1 down‐regulation via shRNA inhibits the growth of pancreatic adenocarcinoma xenografts. J Cell Mol Med. 2012;16(11):2690‐2700. doi:10.1111/j.1582‐4934.2012.01587.x 22568538 10.1111/j.1582-4934.2012.01587.x PMC3435473

[33] Wang YH , Cheng TY , Chen TY , et al. Plasmalemmal vesicle associated protein (PLVAP) as a therapeutic target for treatment of hepatocellular carcinoma. BMC Cancer. 2014;14:815. doi:10.1186/1471‐2407‐14‐815 25376302 10.1186/1471-2407-14-815 PMC4233082

[34] Huang Y , Goel S , Duda DG , et al. Vascular normalization as an emerging strategy to enhance cancer immunotherapy. Cancer Res. 2013;73(10):2943‐2948. doi:10.1158/0008‐5472.CAN‐12‐4354 23440426 10.1158/0008-5472.CAN-12-4354 PMC3655127

[35] Matsuzaki H , Kai K , Komohara Y , et al. Abnormal vessels potentially accelerate glioblastoma proliferation by inducing the protumor activation of macrophages. Cancer Sci. 2025;116(4):897‐909. 39921277 10.1111/cas.70014 PMC11967248

[36] Goumans MJ , Valdimarsdottir G , Itoh S , et al. Activin receptor‐like kinase (ALK)1 is an antagonistic mediator of lateral TGFbeta/ALK5 signaling. Mol Cell. 2003;12(4):817‐828. doi:10.1016/S1097‐2765(03)00386‐1 14580334 10.1016/s1097-2765(03)00386-1

[37] Madri JA , Pratt BM , Tucker AM . Phenotypic modulation of endothelial cells by transforming growth factor‐beta depends upon the composition and organization of the extracellular matrix. J Cell Biol. 1988;106(4):1375‐1384. doi:10.1083/jcb.106.4.1375 3283153 10.1083/jcb.106.4.1375 PMC2115017

[38] Liang L , Chai C , Liu A , et al. Single‐cell transcriptome analysis reveals reciprocal epithelial and endothelial cell evolution in ovarian cancer. iScience. 2024;27(12):111417. doi:10.1016/j.isci.2024.111417 39717089 10.1016/j.isci.2024.111417 PMC11665315

[39] Marchetti GM , Burwell TJ , Peterson NC , et al. Targeted drug delivery via caveolae‐associated protein PV1 improves lung fibrosis. Commun Biol. 2019;2:92. doi:10.1038/s42003‐019‐0337‐2 30854484 10.1038/s42003-019-0337-2 PMC6405929

[40] Myerson JW , Braender B , Mcpherson O , et al. Flexible nanoparticles reach sterically obscured endothelial targets inaccessible to rigid nanoparticles. Adv Mater. 2018;30(32):e1802373. doi:10.1002/adma.201802373 29956381 10.1002/adma.201802373 PMC6385877

[41] Bosma EK , Van Noorden CJF , Schlingemann RO , et al. The role of plasmalemma vesicle‐associated protein in pathological breakdown of blood‒brain and blood‒retinal barriers: potential novel therapeutic target for cerebral edema and diabetic macular edema. Fluids Barriers CNS. 2018;15(1):24. doi:10.1186/s12987‐018‐0109‐2 30231925 10.1186/s12987-018-0109-2 PMC6146740

[42] Nakagami Y , Hatano E , Chayama Y , et al. An anti‐PLVAP antibody suppresses laser‐induced choroidal neovascularization in monkeys. Eur J Pharmacol. 2019;854:240‐246. doi:10.1016/j.ejphar.2019.04.035 31026444 10.1016/j.ejphar.2019.04.035

[43] Chen BB , Shih TT . DCE‐MRI in hepatocellular carcinoma‐clinical and therapeutic image biomarker. World J Gastroenterol. 2014;20(12):3125‐3134. doi:10.3748/wjg.v20.i12.3125 24695624 10.3748/wjg.v20.i12.3125 PMC3964384

[44] Novitskiy SV , Pickup MW , Gorska AE , et al. TGF‐β receptor II loss promotes mammary carcinoma progression by Th17 dependent mechanisms. Cancer Discov. 2011;1(5):430‐441. doi:10.1158/2159‐8290.CD‐11‐0100 22408746 10.1158/2159-8290.CD-11-0100 PMC3297196

[45] Bierie B , Chung CH , Parker JS , et al. Abrogation of TGF‐beta signaling enhances chemokine production and correlates with prognosis in human breast cancer. J Clin Invest. 2009;119(6):1571‐1582. doi:10.1172/JCI37480 19451693 10.1172/JCI37480 PMC2689133

[46] Toonkel RL , Borczuk AC , Powell CA . Tgf‐beta signaling pathway in lung adenocarcinoma invasion. J Thorac Oncol. 2010;5(2):153‐157. doi:10.1097/JTO.0b013e3181c8cc0c 20101143 10.1097/JTO.0b013e3181c8cc0c PMC2992959

