# Supplementary material for: PLVAP mediates the regulation of the tumour microenvironment in early‐stage lung adenocarcinoma
Source: Clin Transl Med. 2025 Dec 22;15(12):e70532. doi: 10.1002/ctm2.70532 (PMC12723075; doi:10.1002/ctm2.70532)
Supplement: Supplementary file 3 — Supporting Information [file CTM2-15-e70532-s001.docx]

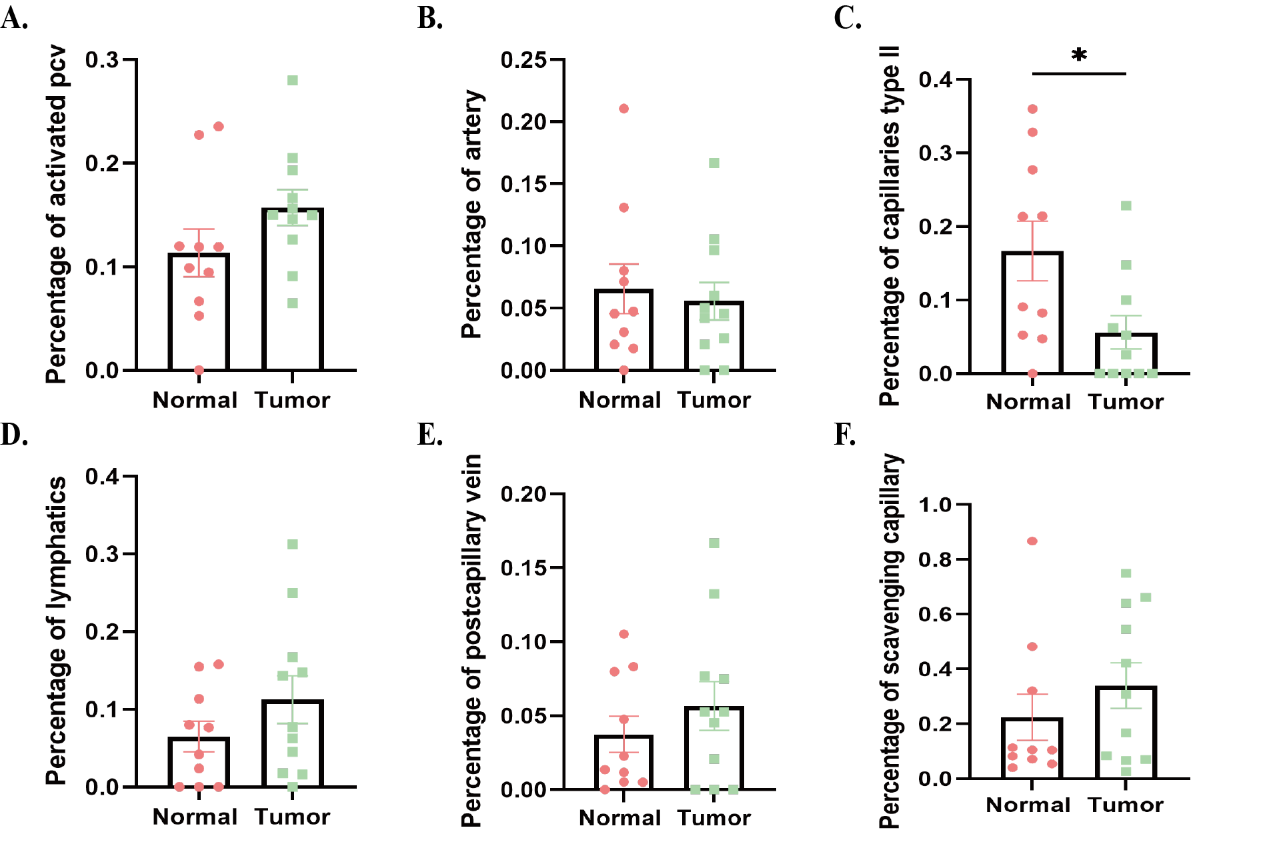


Figure S1: Percentage of endothelial cell subpopulations in each group.


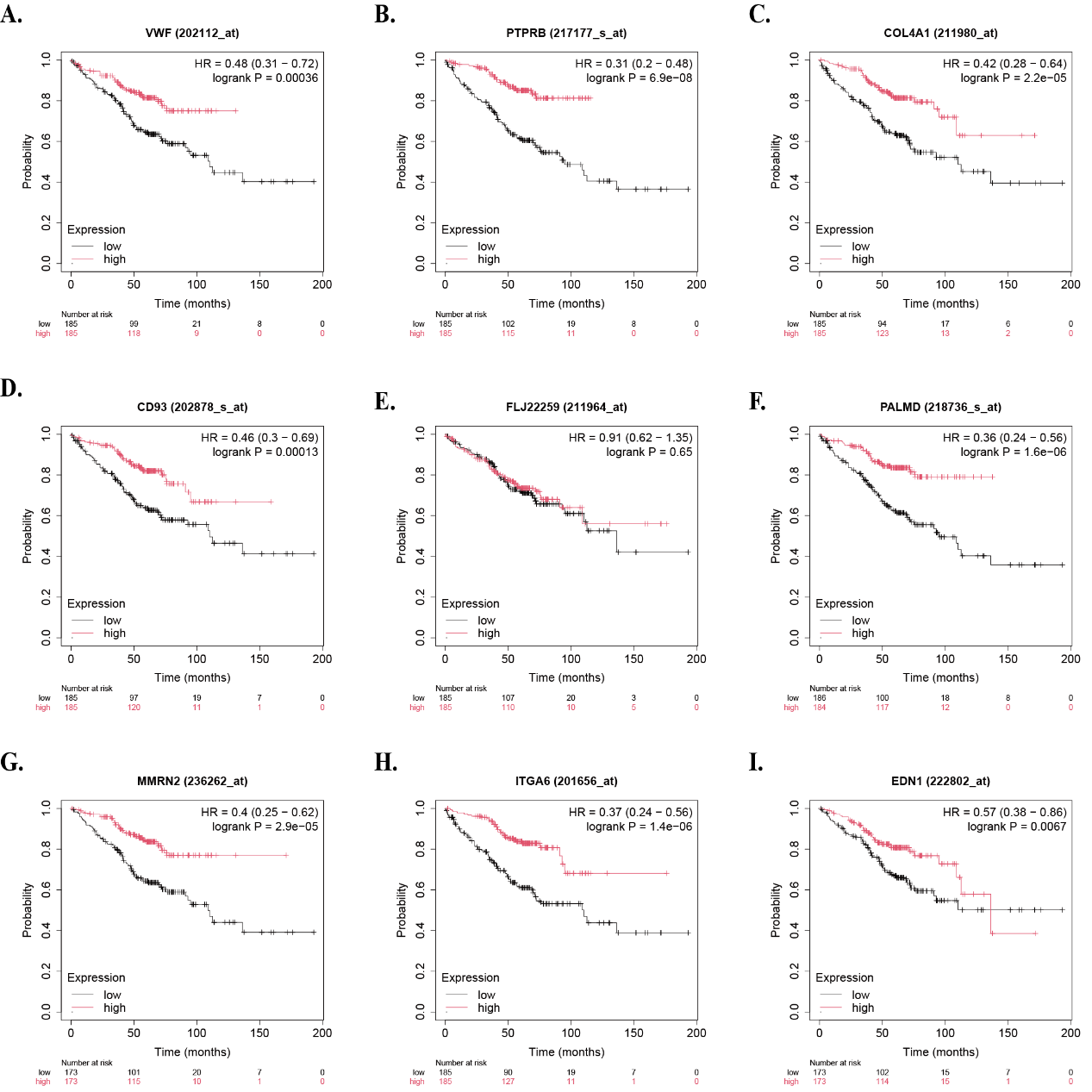


Figure S2: Filtering for survival prognosis of signature genes.
